# Quantitative mechanistic model reveals key determinants of placental IgG transfer and informs prenatal immunization strategies

**DOI:** 10.1101/2023.04.18.537285

**Published:** 2023-04-19

**Authors:** Remziye Rachel Erdogan, Sepideh Dolatshahi

**Affiliations:** 1 Department of Biomedical Engineering, University of Virginia School of Medicine, Charlottesville, VA, 22908; 2 Carter Immunology Center, University of Virginia School of Medicine, Charlottesville, VA, 22908

## Abstract

Transplacental antibody transfer is crucially important in shaping neonatal immunity. Recently, prenatal maternal immunization has been employed to boost pathogen-specific immunoglobulin G (IgG) transfer to the fetus. Multiple factors have been implicated in antibody transfer, but how these key dynamic regulators work together to elicit the observed selectivity is pertinent to engineering vaccines for mothers to optimally immunize their newborns. Here, we present the first quantitative mechanistic model to uncover the determinants of placental antibody transfer and inform personalized immunization approaches. We identified placental FcγRIIb primarily expressed by endothelial cells as a limiting factor in this receptor-mediated transfer, which plays a key role in promoting preferential transport of subclasses IgG1, IgG3, and IgG4, but not IgG2. Integrated computational modeling and *in vitro* experiments reveal that IgG subclass abundance, Fc receptor (FcR) binding affinity, and FcR abundance in syncytiotrophoblasts and endothelial cells contribute to inter-subclass competition and potentially inter- and intra-patient antibody transfer heterogeneity. We utilize this model as an *in silico* immunization testbed, unveiling an opportunity for precision prenatal immunization approaches that account for a patient’s anticipated gestational length, vaccine-induced IgG subclass, and placental FcR expression. By combining a computational model of maternal vaccination with this placental transfer model, we identified the optimal gestational age range for vaccination that maximizes the titer of antibody in the newborn. This optimum vaccination time varies with gestational age, placental properties, and vaccine-specific dynamics. This computational approach provides new perspectives on the dynamics of maternal-fetal antibody transfer in humans and potential avenues to optimize prenatal vaccinations that promote neonatal immunity.

## Introduction

Neonates are vulnerable to infections due to their tolerogenic immune phenotype; for the same reason, neonatal vaccinations have been met with limited success to date (1). To provide passive immunity while the neonatal immune system adapts to the environment *ex utero*, maternal immunoglobulin G (IgG) is selectively transferred across the placenta during gestation. Not only do these maternal antibodies confer neonates with passive early life immunity against infectious diseases, but also they shape the trajectory of neonatal immune development by priming the cellular immune response (2–4)^,^. Despite this crucial role, the molecular underpinnings of IgG transplacental transport are incompletely described to date.

Though the placenta obstructs maternal-fetal transport of most large molecules, maternal IgG is selectively transported by way of Fc receptor (FcR)-mediated transcytosis. To enter fetal circulation, IgG must undergo transcytosis through two key cellular layers of the placenta: syncytiotrophoblasts (STBs) and endothelial cells (ECs). The neonatal Fc receptor (FcRn) is known to mediate IgG transport through STBs, but whether this mechanism is sufficient for IgG transport through ECs remains unclear (5–7). In addition to FcRn, STBs and ECs express several Fc gamma receptor (FcγR) isoforms with heterogeneous affinity for IgG (8–10). These classical FcγRs may contribute selective placental “sieving” of IgG dependent upon fragment crystallizable (Fc) characteristics, e.g., IgG subclass and N-linked glycosylation (11–14), yet there is conflicting evidence surrounding the involvement of classical FcγRs in IgG transport (9,10,15,16). Recent studies hypothesized a role for STB-bound FcγRIIIa or EC-bound FcγRIIb in IgG transfer (9,12,17–20), yet others showed FcRn is sufficient for IgG transport in isolated placental ECs *in vitro* and in mouse placenta *in vivo* (14,21,22). Though informative, the experimental methods underlying these findings do not capture the complexity or time scale of placental antibody transport. To disentangle the involvement of non-canonical FcγRs in IgG sieving, innovative methods that recapitulate the longitudinal dynamics of this fundamental process are needed.

Placental antibody transfer has been leveraged by maternal prenatal vaccines which boost pathogen-specific IgG transport to the neonate (23). Identifying the ideal immunization strategy to maximize neonatal antibody titers is non-trivial due the growth and development of the placenta which dynamically regulates IgG transport efficiency. These complex dynamics coupled with known maternal immune adaptations during pregnancy define a unique immunization design space which poses a challenge to empirical vaccine optimization in clinical trials alone. Predictive kinetic-dynamic modeling can be leveraged to efficiently design vaccines that maximize IgG transfer to the neonate. The ability to make *in silico* predictions of vaccine-induced antibody transfer would enable pre-clinical dosing strategy studies, expediting translation from bench to bedside.

In the United States, expecting mothers are routinely vaccinated against tetanus, diphtheria, and acellular pertussis (Tdap) during the early third trimester (24), but emerging evidence suggests that this recommendation may not optimally protect the entire population. First, it has been shown that maternal Tdap immunization earlier in gestation results in higher pertussis toxin (PT)- and filamentous hemagglutinin (FHA)-IgG in infants regardless of gestational length (24–26). Second, current vaccination efforts are designed to elicit high antibody transfer to term neonates, yet third trimester immunization may not allot sufficient time for the mother to mount a humoral immune response and transfer antibodies to preterm neonates. Collectively, this evidence supports the potential for personalized vaccine approaches to maximize pathogen-specific IgG transfer to the neonate, especially among premature neonates.

To uncover the molecular regulators of placental antibody transport and to inform the development of novel, personalized immunization approaches, we developed the first computational model of placental IgG transfer. We use this model as an *in silico* testbed for prenatal vaccine design and identify potential strategies to improve transfer of vaccine-induced antibodies, both at a patient-specific and population level. Ultimately, this model-driven investigation sheds light on the dynamic regulation of maternal-fetal IgG transfer and provides a foundation to develop precision vaccine approaches which promote neonatal immunity.

## Results

### Mechanistic model recapitulates IgG subclass-specific placental transfer

**Figure 1.**
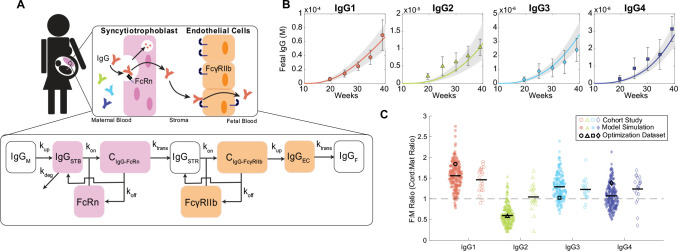
Mechanistic model captures IgG subclass-specific transfer dynamics. (A) The schematic depicts the ODE model representing transport of IgG through the placenta, mapped onto a graphical representation of the maternal-fetal interface. Reaction rate constant associated with each process is depicted on the corresponding arrow. Each square represents a molar concentration in each compartment. “C” denotes complexes of bound IgG and Fc receptor either in STB endosomes (C_IgG-FcRn_) or at the EC surface (C_IgG-FcγRIIb_). For simplicity, the schematic represents only bulk IgG transport, whereas the model includes separate equations for each IgG subclass. (B) Simulated fetal IgG subclass levels vs. time (lines) are plotted overlaid with data used for parameter estimation. The overlaid data are the mean and standard deviation of IgG subclasses in the umbilical cord between 17–41 weeks gestation (n = 107) measured using cordocentesis. Gray lines represent 500 perturbation simulations resulting from sampling parameters from the ranges determined by CaliPro. (C) The swarm charts show the fetal/maternal ratio (F:M ratio) for each IgG subclass at 40 weeks simulation time resulting from 500 simulations sampling from the optimized range of parameters (filled symbols). Each symbol represents a single simulation. The bold symbols (black outline) represent the mean of IgG subclass cord/maternal transfer ratio at parturition from the human study used during parameter optimization. The swarm charts (hollow symbols) show the mean transfer ratio for each IgG subclass compiled across cohort studies mined from the literature. Black line depicts the sample mean. Dashed line at F:M (cord/maternal) ratio = 1 indicates equal concentration in maternal and fetal blood at parturition.

To elucidate mechanisms of IgG transfer and selective “sieving”, we devised a dynamic model of IgG transplacental transfer. The model consists of ordinary differential equations (ODEs) describing IgG mass transport through the distinct layers comprising the maternal-fetal interface: STB FcRn-mediated transcytosis, diffusion through intervillous stroma, and EC FcγRIIb-mediated transcytosis (Fig 1A) (see Appendix S1 for model equations). FcRn is widely implicated in placental IgG transport; we additionally chose to incorporate FcγRIIb into the model due to its expression in term placental ECs and demonstrated role in EC transcytosis *in vitro* (9,19,27). Model parameters were derived from literature when available, and unknown parameters were estimated by fitting predictions of fetal IgG to measurement data obtained from cordocentesis in human pregnancies (Fig 1B, Table 1). Parameter estimation was performed using CaliPro as described elsewhere (28,29) (see Methods). With this optimized parameter set, the model recapitulated dynamics of both bulk and subclass-specific IgG transfer, predicting the IgG subclass transfer hierarchy most frequently reported in literature (IgG1 > IgG3 > IgG4 > IgG2) (Fig 1C, S1 Fig) (11,29,30).

### Transcytosis rate parameters and Fc receptor expression regulate IgG transfer efficiency and selectivity

**Fig 2.**
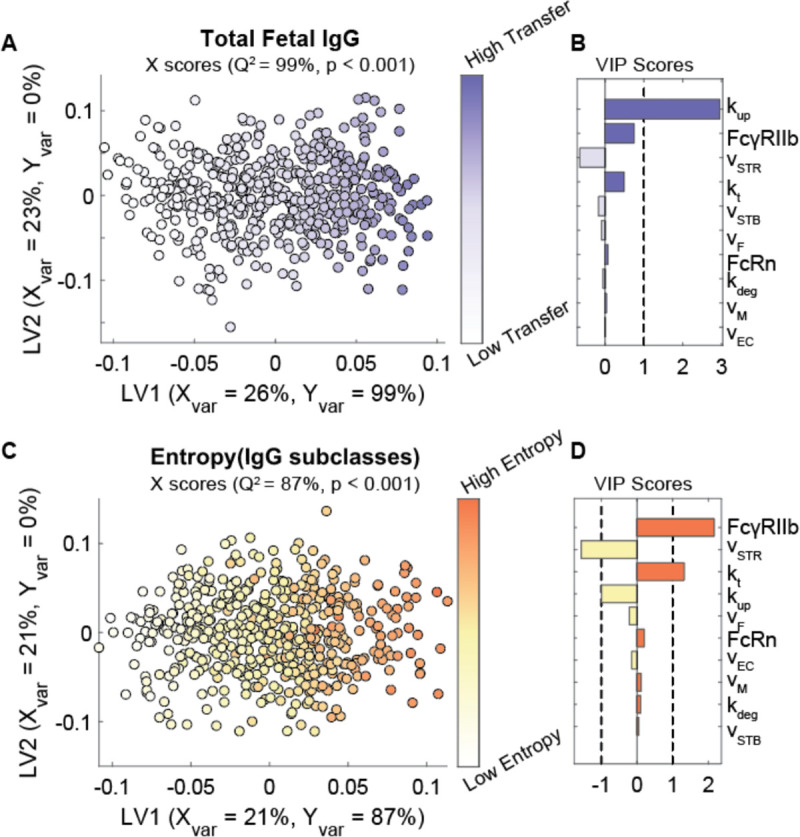
Key parameters associated with transcytosis mechanism and Fc receptor expression regulate IgG transfer. (A-B) An OPLSR model was built to determine parameters predictive of total fetal IgG. (A) The scores plot showing X scores on latent variables 1 and 2 (LV1 and LV2) of simulated fetal IgG concentration at parturition for 500 perturbation parameter sets. Each circle represents a single perturbation experiment colored proportional to the concentration of IgG in the fetus at delivery. The model was orthogonalized such that the direction of maximum variance is in the direction of latent variable 1 (LV1), capturing 99% of the Y variation. Conversely, LV2 captures the variability in parameters that do not contribute to increased IgG transfer (Y variation = 0%). (B) The bar graph depicts the variable importance in projection (VIP) scores, which quantifies the contribution of each parameter to total fetal IgG for a term delivery. VIP scores are artificially oriented in the direction of their loadings on LV1. VIP score > 1 indicates a greater-than-average influence on the model. The model performed with high predictability power (Q^2^ = 99%). The model had a mean squared error which outperformed 1000 random models with permutated labels (p < 0.001). (C-D) An analogous OPLSR model was built to predict entropy among fetal IgG subclass levels at the end of gestation. (C) The scores plot showing X scores on LV1 and LV2 of simulated fetal IgG concentration at parturition for 500 perturbation parameter sets where each circle represents a single perturbation experiment colored proportional to the computed entropy among IgG subclasses in the fetus at delivery. The model was orthogonalized such that LV1 captures features associated with the highest entropy, accounting for 87% of the Y variation. Conversely, LV2 captures the variability in parameters that do not contribute to increased entropy (Y variation = 0%). (D) The VIP scores bar plot shows the contribution of each parameter to the entropy of IgG subclasses in fetal blood for a term delivery. VIP scores are artificially oriented in the direction of their loadings on LV1. VIP score > 1 indicates a greater-than-average influence on the model. The model performed with high predictability power (Q^2^ = 87%). The model had a low mean squared error which outperformed 1000 models based on randomly permutated labels (p < 0.001).

To determine the contribution of each parameter to the subclass-specific dynamics predicted by the model, we performed global sensitivity analyses. We generated 500 perturbed parameter sets using Latin Hypercube Sampling (LHS) from uniform distributions spanning the maximum likelihood parameter ranges determined by CaliPro (28,33). For each parameter set, the predicted fetal IgG at 40 weeks was calculated. To determine the parameters with the greatest influence on total IgG transfer (Fig 2A–B) and on differential transfer between IgG subclasses defined as entropy of subclasses (Fig 2C–D), orthogonalized partial least squares regression (OPLSR) models were developed (34). Variable importance in projection (VIP) scores revealed that total IgG transfer was most sensitive to the rate of IgG uptake by STBs and ECs (k_up_) and FcγRIIb expression (Fig 2B). The volume of interstitial stroma (v_STR_) and fetal blood volume (v_f_) negatively correlated with IgG transfer, likely because increasing the volume of either compartment dilutes the IgG concentration. To reveal parameters that contribute to the difference in IgG transfer among IgG subclasses, we built a separate OPLSR model predicting the entropy among IgG subclasses (Methods) (Fig 2C–D). Interestingly, VIP score analysis showed FcγRIIb contributes most to the differential transfer rates among IgG subclasses. FcγRIIb is a low-affinity IgG receptor with highest affinity for subclasses IgG3 and IgG4, suggesting that it constitutes a rate-limiting factor of IgG transfer which fine-tunes the subclass transfer hierarchy. The rate of IgG transcytosis across cells (k_t_) also contributes to subclass-specific transfer; this is in part because k_t_ acts in an FcR-dependent manner, further amplifying the effects of Fc-mediated selectivity. Thus, the rate of cargo internalization and fetoplacental size drives the concentration of bulk IgG in the fetus, whereas FcRn and FcγRIIb collaborate to fine-tune patterns of IgG subclass-specific transfer.

### Endothelial cell-bound FcγRIIb is a key driver of subclass-specific IgG transfer

**Figure 3.**
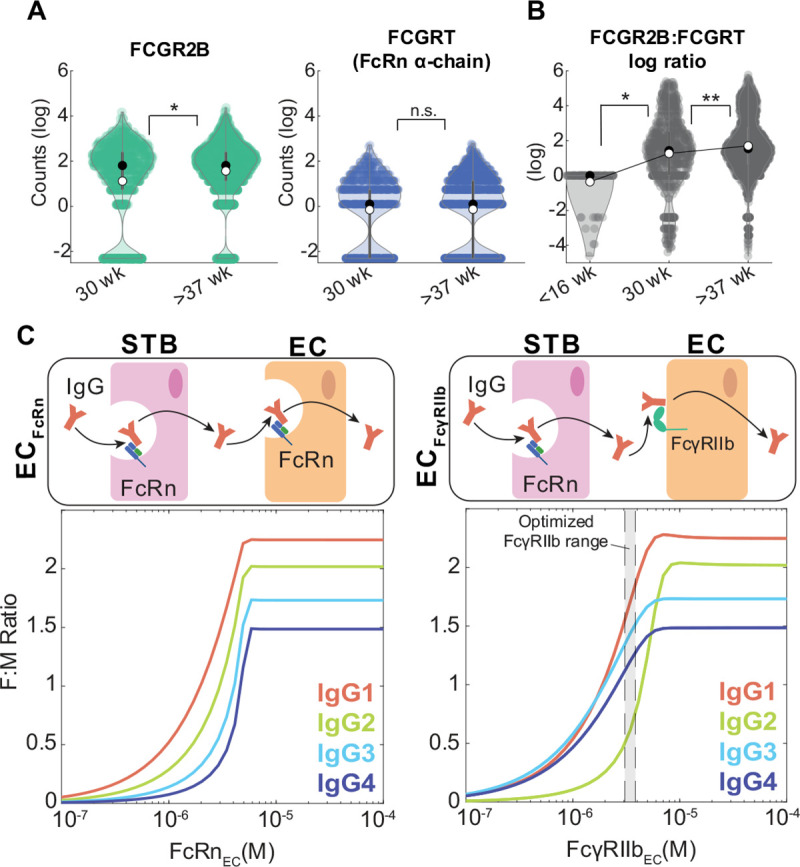
Endothelial cell-bound FcγRIIb is a key driver of subclass-specific IgG transfer. (A) Violin plots show *FCGR2B* and *FCGRT* transcript levels in preterm, preeclamptic ECs (30 weeks) compared to term, healthy ECs (>37 weeks) from single cell RNA sequencing data. Black circles represent the sample median, white circles represent the sample mean. (B) Violin plots show the log-transformed ratio of *FCGR2B* to *FCGRT* counts in ECs from single cell RNA seq data from two separate cohorts spanning 3 gestational time points: <16 weeks, 30 weeks, and >37 weeks. Black circles represent the sample median, white circles represent the sample mean. (C) Model-simulated IgG subclass transfer ratios as a function of EC Fc receptor expression with ECs expressing only FcRn (left, EC_FcRn_) or FcγRIIb (right, EC_FcγRIIb_). The corresponding schematics depict the simulation conditions. The shaded region shows the optimized range of FcγRIIb expression as determined by parameter optimization. Statistical significance in (B-C) was determined by a Wilcoxon rank sum test (* p < 0.01, ** p < 0.001, n.s. not significant).

It is well-established that placental IgG transport increases during the third trimester in humans, so we asked whether increasing FcγRIIb or FcRn expression in ECs drives increased transport efficiency. To gain insight into the expression dynamics of the genes coding for FcγRIIb and FcRn, namely *FCGR2B* and *FCGRT* (FcRn heavy chain), in fetal ECs across gestation, we analyzed two single cell RNA sequencing (scRNA-seq) data sets from published literature spanning three gestational time points: (1) first trimester samples from elective abortions (< 12 weeks), (2) early third trimester samples from preeclamptic cesarean sections (30 weeks), and (3) late third trimester samples from uncomplicated cesarean sections (38 weeks) (35,36). Interestingly, *FCGR2B* expression was significantly higher in late compared to early third trimester ECs, but we did not detect a significant difference in *FCGRT* expression between early and late third trimester ECs (Fig 3A). Conversely, *FCGRT* but not *FCGR2B* transcripts were detected in first trimester ECs, and the ratio of *FCGR2B* to *FCGRT* expression per cell increased across time points (Fig 3B, S2 Fig), suggesting that FcγRIIb dynamically regulates IgG transcytosis in ECs.

To assess the contribution of endothelial cell FcγRIIb to IgG transport, we focused on the role of FcγRIIb as a determinant of subclass-specific transfer. Previous studies found that FcRn and FcγRIIb were co-expressed by placental ECs (9,37), and FcRn has a noticeably higher affinity for all IgG subclasses (Table 1). This raises the question of whether FcRn alone is responsible for IgG transfer across fetal ECs. As FcγRIIb binds subclasses IgG1, IgG3, and IgG4 with greater affinity than IgG2, we hypothesized that FcγRIIb is necessary to predict preferential transfer of IgG1, IgG3, and IgG4 over IgG2. To test this hypothesis, we compared model-predicted fetal-maternal IgG ratios from simulations assuming ECs solely express FcRn (EC_FcRn_) or FcγRIIb (EC_FcγRIIb_). The EC_FcRn_ model did not capture the preferential transfer of IgG1, IgG3, and IgG4 at any FcRn expression level (Fig 3C, left). Interestingly, in the EC_FcγRIIb_ model the subclass transfer hierarchy shifted from IgG3 > IgG1 > IgG4 > IgG2 to IgG1 > IgG2 > IgG3 > IgG4 with increasing FcγRIIb expression (Fig 3C, right). As the transcriptome analysis demonstrated that *FCGRT* and *FCGR2B* can be co-expressed in fetal ECs, we questioned whether this FcγRIIb-dependent mechanism of subclass-specific transfer is present when FcRn and FcγRIIb are co-expressed (EC_FcRn,FcγRIIb_). In a model where ECs express both FcRn and FcγRIIb, we found that the predicted subclass transfer hierarchy was IgG1 > IgG3 > IgG4 > IgG2 when FcRn expression was less than 40% of FcγRIIb (S3 Fig), demonstrating that higher relative expression of FcγRIIb to FcRn by ECs drives subclass-specific IgG placental transfer. Together, these data implicate FcγRIIb in ECs as a key regulator of antibody transfer dynamics across gestation.

### IgG subclasses compete for receptor-mediated transcytosis

**Fig 4.**
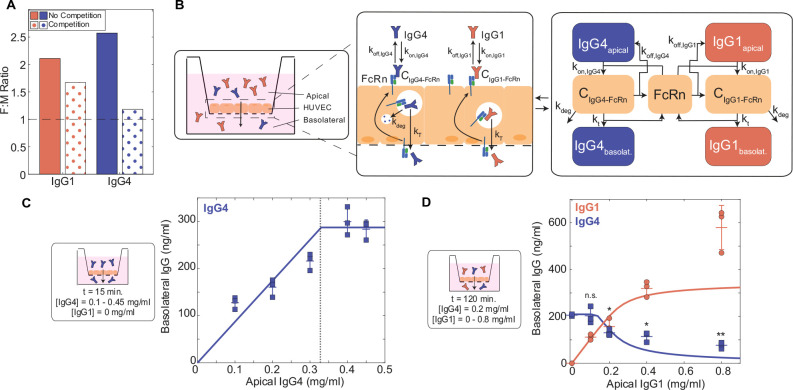
IgG1 competes with Fc IgG4 transcytosis in ECs. (A) The bar plot shows the effect of mixing IgG subclasses together simulated in the compartmental placental model. Solid filled bars represent IgG1 and IgG4 isolated (no competition), and patterned bars represent IgG1 and IgG4 mixed (competition). (B) Schematic showing a cross-sectional view of the Transwell system and corresponding mechanistic model representation of FcRn-mediated IgG4 and IgG1 transport through HUVEC. Right panel: Arrows indicate the direction of transfer (apical to basolateral). Center panel and right panel: Reaction rate constant associated with each process is depicted on the corresponding arrow. (C) Model-predicted IgG4 transcytosis (y-axis) with increasing apical concentration (x-axis) (solid line) is plotted against experimental data. The corresponding experimental conditions are displayed in the panel on the left. Experimental results of IgG1 and IgG4 transcytosis in HUVEC in the Transwell assay system from three independent replicates are shown with their mean and standard deviation. The dashed line represents the model-predicted and experimentally validated transcytosis saturation point. (D) Model-predicted IgG4 and IgG1 transcytosis (y-axis) with increasing apical concentration of IgG1 (x-axis) (solid lines) is plotted against experimental data. The corresponding experimental conditions are displayed in the panel on the left. Experimental results of IgG1 and IgG4 transcytosis in HUVEC in the Transwell assay system from three independent replicates are shown with their mean and standard deviation. The dashed line represents the model-predicted and experimentally validated transcytosis saturation point. Statistical comparisons shown are between IgG4 only (far left graph) in the absence of IgG1 and all combinations of IgG1 and IgG4 mixed using a one-tailed two-sample t-test (* p < 0.05, ** p < 0.001, n.s. not significant).

IgG subclass abundances vary across individuals, which may contribute to patient-specific differences in IgG subclass transfer. On the other hand, our finding that FcγRIIb limitation plays a key role in determining the subclass transfer hierarchy suggests that maternal IgG subclasses compete for receptor-mediated transcytosis. In agreement with this hypothesis, the model predicted that all four IgG subclasses transferred less efficiently when mixed compared to each subclass in isolation of the others (Fig 4A), and the effect of mixing was inversely proportional to subclass abundance in maternal serum at low FcγRIIb expression levels (S4 Fig). To further elucidate the role of inter-subclass competition in determining transport efficiency, we explored the effect of IgG1 on IgG4 transcytosis in ECs. We chose to model IgG1 and IgG4 as a case study because they have the highest and lowest abundance in maternal serum, respectively, yet both generally exhibit a cord/maternal ratio > 1 despite their different abundances. Based on a simple kinetic reaction model, the inter-subclass competition can be described by the following closed-form equation:

$$C_{IgG4-FcRn}=\frac{FcRn_{0}[IgG4]}{K_{D,IgG4}(1\;+\;\frac{\left[IgG4\right]}{K_{D,IgG4}}\;+\;\frac{\left[IgG1\right]}{K_{D,IgG1}})}$$

Where $C_{I\text{gG4-FcRn}\;}$ is the concentration of bound IgG⁡4 and FcRn, ${FcRn}_{0}$ is the total concentration of receptor, [IgG1] and [IgG4] are concentrations of maternal IgG1 and IgG4, and $K_{D,IgG1}$ and $K_{D,IgG4}$ are their respective dissociation constants (see derivation in Methods). Intuitively, $C_{IgG4\text{-}FcRn}$ is a function of IgG1 abundance and affinity for FcRn, such that either increasing [IgG1] or decreasing $K_{D,IgG1}$ (i.e., increasing IgG1-FcRn affinity) decrease $C_{IgG4-FcRn}$ and consequently, IgG4 transcytosis.

To validate and further characterize this hypothesized competition, we used an *in vitro* system to model human IgG transcytosis through human umbilical vein endothelial cells (HUVEC) cultured on Transwell permeable membranes (Fig 4B) (Methods). In parallel, we devised a mechanistic model of receptor-mediated transcytosis and optimized its parameters by fitting to temporal data of IgG4 transcytosis in the HUVEC experimental system ([IgG4_apical_] = 0.5 or 0.125 mg/ml) (S5 Fig, Table 2). To recapitulate the physiological setting where FcγRIIb receptor is limiting similar to the physiological conditions, we varied IgG4 levels to determine the range where the transferred IgG4 stopped increasing with increasing apical IgG4 levels. Although HUVECs express FcRn but not FcγRIIb, our findings remain valid because the affinities of IgG1 and IgG4 for either receptor are on the same order of magnitude and thus the dynamics of subclass competition are comparable. Our mechanistic model predicted that IgG4 transcytosis saturates at [IgG4_apical_] > 0.33 mg/ml, indicating FcRn limitation; we validated this finding in an analogous *in vitro* experiment in the Transwell system (Fig 4C). To quantify the effect of IgG1 on IgG4 transcytosis, we simulated the effect of increasing IgG1 concentration when IgG4 was fixed at a concentration just below the FcRn saturation point ([IgG4_apical_] = 0.2 mg/ml) (Fig 4C). Our model predicted that IgG4 transcytosis would not be affected by the presence of IgG1 until the total IgG concentration exceeded the saturation point—that is, [IgG1_apical_] + [IgG4_apical_] > 0.33 mg/ml. In agreement with our computational model prediction, increasing IgG1 caused IgG4 transcytosis to decrease only when the total IgG concentration exceeded the FcRn saturation point (Fig 4C). IgG subclass-specific transfer is thus a function of subclass abundances and FcR affinity, and competition for FcR-mediated transcytosis occurs when FcR expression is limited.

### Optimal Tdap vaccine schedules depend on gestational age and patient-specific Fc receptor expression

**Figure 5.**
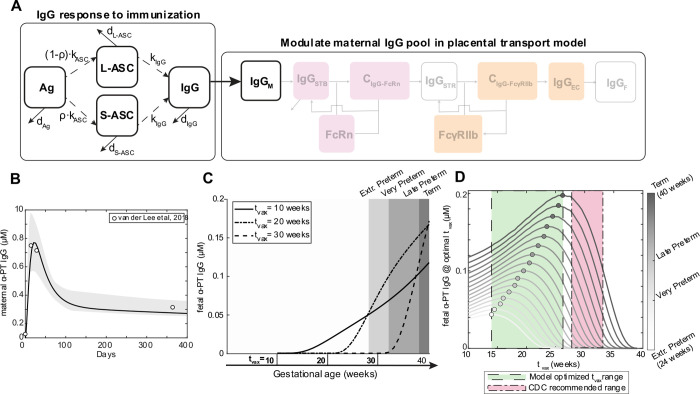
Optimal Tdap immunization time is gestational age dependent. (A) Schematic shows mechanistic model of maternal plasma cell antibody secretion post-vaccination. Vaccine-induced maternal IgG kinetics are integrated into the maternal compartment of the placental IgG transport model, shown in the righthand box. Dashed arrows indicate regulatory processes, solid lines represent processes modeled using mass action kinetics. L_ASC_: Long-lived antibody secreting cell, S_ASC_: short-lived antibody-secreting cell, Ag: antigen. (B) Simulated maternal anti-pertussis IgG concentrations vs. time is plotted with a solid line and overlaid with measured IgG concentrations following Tdap vaccination in women of childbearing age from published literature (n = 105). The 95% confidence intervals are shown with light gray shading. (C) Simulated fetal anti-pertussis toxin IgG concentrations are plotted against time following maternal vaccination at three sample vaccination times (indicated along the x-axis): t_vax_ = 10, 20, and 30 weeks gestational age. Shaded regions correspond to gestational length groups (left to right): extremely preterm, very preterm, late preterm, term. (D) Simulated anti-pertussis toxin IgG in the fetus at the time of delivery (y-axis) is plotted over a range of simulated vaccination times (x-axis) and gestational ages (color intensity, color bar on right). The optimal vaccination time and corresponding fetal IgG levels are marked with a circle. The current CDC-recommended maternal vaccination window is highlighted in red, and the model-predicted optimal vaccination range encompassing all gestational age groups is highlighted in green.

Having uncovered new insights into the dynamic regulation of antibody transfer by FcRs, we sought to uncover key features influencing the transfer of vaccine-induced antibodies which could be considered in precision immunization approaches. To this end, we developed an *in silico* immunization testbed by augmenting the placental antibody transfer model with simulated kinetics of the maternal antibody response post-immunization (Fig 5A). As a case study of a maternal vaccine currently in use, we adapted an existing model of plasma cell activation and antibody secretion following malaria infection to recapitulate IgG responses in women of child-bearing age following Tdap immunization (Methods) (Fig 5B) (38,39). A dose of antigen representing maternal immunization was applied to the system at three representative gestational ages t_vax_ = 10, 20, and 30 weeks to stimulate antibody secreting cell proliferation and anti-pertussis toxin IgG (α-PT IgG) secretion and transfer to the fetus (Fig 5C). Though the correlate of protection for pertussis infection still remains elusive, some possible roles of IgG in pertussis infection include neutralization and opsonophagocytosis (40,41). For the purposes of this study, we assumed that neutralizing antibodies confers protection against pertussis toxin in the neonate and therefore the optimal t_vax_ is defined as the gestational age at immunization resulting in the highest α-PT IgG titer in the fetus at birth. Interestingly, the interplay of the immunization time and placenta transfer results in complex temporal changes in fetal IgG levels, emphasizing the importance of model simulations in predicting the optimal t_vax_. For example, immunization at t_vax_ = 20 weeks results in higher fetal IgG levels compared to 10 and 30 weeks for very preterm, preterm and term pregnancies (Fig 5C). We then ran *in silico* simulations over a range of possible immunization times to determine the optimal t_vax_ for newborns born at different weeks of gestation (Fig 5D).

Empirical studies have shown that earlier vaccination may be an effective strategy to protect preterm neonates who typically receive a lower quantity of maternal antibodies (24–26). Using our immunization testbed, we first asked how the optimal Tdap administration schedule varies for the average patient delivering at different gestational time points (i.e., term, late preterm, very preterm, or extremely preterm). In the case of full term gestation, the model-predicted optimal t_vax_ was during the 26^th^ week of gestation, but Tdap immunization as early as the 10^th^ week still resulted in two-thirds of the optimal IgG concentration being transferred to term newborns (Fig 5D). Optimal t_vax_ scaled with increasing gestational length, suggesting that risk for preterm birth should be considered when determining prenatal immunization schedules (Fig 5D). Strikingly, we found the optimal t_vax_ fell during the second trimester (between 14–26 weeks) regardless of preterm birth status, which is earlier than the window recommended by the Centers for Disease Control and Prevention (CDC) (28–34 weeks) and in agreement with studies reporting higher titer of pertussis antibodies in the fetus following maternal vaccination during second trimester as opposed to third trimester (24,26,43). Thus, optimal immunization schedules are gestational length-dependent, but a specific vaccination window of opportunity during gestation could be an effective population-level strategy to ensure coverage for preterm and term newborns alike.

**Figure 6.**
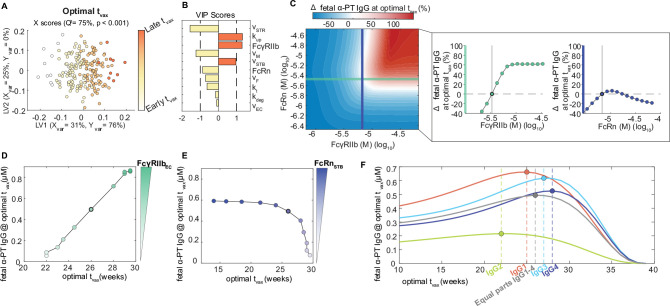
Optimal vaccination schedules depend on patient-specific placental and maternal parameters. (A-B) The OPLSR model predicts the optimal immunization time (t_vax_, weeks gestational age) from perturbed parameter sets. (A) The scores plot showing X scores on LV1 and LV2 of the optimal t_vax_ for 200 perturbed parameter sets. Each circle represents a single perturbation experiment colored proportionally to the predicted optimal t_vax_. The model was orthogonalized such that LV1 is in the direction of maximum variance, capturing 76% of the Y variation. Conversely, LV2 captures the variability in parameters that do not contribute to increased IgG transfer (Y variation = 0%). (B) The bar plot shows VIP scores that quantify the contribution of each parameter to the optimal t_vax_. VIP scores are artificially oriented in the direction of their loadings on LV1. VIP score > 1 indicates a greater-than-average influence on the model. The model performed with high predictability power (Q^2^ = 75%) and with a mean squared error lower than 1000 models based on randomly permutated labels (p < 0.001). (C) The contour map shows the percent change in anti-pertussis toxin IgG (α-PT IgG) in a fetus delivered at 40 weeks relative to the baseline simulation with varying FcRn and FcγRIIb expression. Blue and red correspond to a decrease or increase in fetal IgG relative to the baseline simulation. FcRn and FcγRIIb are shown on a logarithmic scale. The bold indigo and turquoise lines indicate the optimized FcRn and FcγRIIb expression levels, and the intersection point indicates the baseline simulation using parameters estimated by fitting to the average data. The curves plotted to the right are one-dimensional traces indicated by the bold lines on the two-dimensional contour plot and are indicative of simulations where either FcγRIIb (left) or FcRn (right) are held at their optimized expression level and the other receptor is allowed to vary. The dashed line represents the baseline simulation, and the vertical line shows the concentration of FcγRIIb or FcRn at the baseline simulation. The corresponding circle is marked with a bold outline. (D,E) The optimal t_vax_ and corresponding anti-pertussis toxin IgG at term delivery is given for a range of FcγRIIb (D) and FcRn (E) expression levels below their optimized values. The points are connected to show the trajectory of optimal t_vax_ with increasing FcγRIIb or FcRn. FcγRIIb and FcRn are indicated by color intensity of the markers. The baseline simulation is marked in both graphs with a bold outlined circle. (F) A range of t_vax_ are shown for simulations with different compositions of vaccine-induced IgG subclasses. Simulated conditions were vaccine-induced IgG consisting of entirely IgG1, IgG2, IgG3, or IgG4, or with equal parts IgG1, IgG2, IgG3, and IgG4. The optimal t_vax_ and corresponding IgG titer for a given subclass (or mixture of subclasses) for each condition is marked with a circle and labeled on the x-axis.

Other sources of patient-to-patient variability that could influence optimal immunization schedules include differences in placental transfer efficiency linked to FcR-mediated transcytosis and the quality and quantity of IgG induced by the vaccine. To determine key variables that should be considered in patient-specific immunization approaches, we performed a global sensitivity analysis to uncover parameters most influential on optimal t_vax_. We optimized t_vax_ in simulations from 200 perturbed parameter sets sampled from a uniform distribution plus or minus 10% of each parameter’s optimized value, then built an OPLSR model to predict the optimal t_vax_ given each parameter set (Fig 6A, 6B). Parameters which were associated with later immunization times were the rate of IgG internalization (k_up_), FcγRIIb, and volume of STB endosomes (v_STB_), whereas parameters associated with earlier immunization times were the stromal volume (v_STR_), maternal plasma volume (v_M_), and FcRn (Fig 6B). Intuitively, high maternal and stromal volumes decrease transfer of vaccine-induced antibodies because IgG concentration is diluted in high volume compartments. On the other hand, later vaccination is favorable with increased endosomal volume in STBs and the IgG internalization rate because these parameters increase the overall efficiency of STB transcytosis, facilitating rapid transport of IgG just prior to delivery and minimizing the time IgG is subject to degradation in the fetus whilst *in utero*. Therefore, differences in placental size and function across the population may play a role in determining the ideal patient-specific immunization program.

To investigate the differential impact of FcRn and FcγRIIb on t_vax_ optimization, we simulated immunization at the optimal t_vax_ for term births determined previously (Fig 5D) for mothers with a range of FcRn and FcγRIIb expression. By examining the percent change in pertussis toxin IgG transfer for variations in FcRn and FcγRIIb relative to the average patient, we observed that the greatest increase in α-PT IgG transfer is achieved when both FcRn and FcγRIIb expression levels are high, but IgG transfer decreased when either FcRn or FcγRIIb expression decreased (Fig 6C). To determine whether patients with a deficiency in FcRn or FcγRIIb would benefit from a different immunization schedule than the average patient, we optimized t_vax_ at FcRn and FcγRIIb levels below their average values. As seen in the OPLSR analysis in (Fig 6A–B), this confirmed that high FcγRIIb expression corresponds to later optimal t_vax_, whereas high FcRn expression corresponds to earlier optimal t_vax_ (Fig 6D, 6E). Underlying this disparate role of FcRn and FcγRIIb in vaccine optimization, we found that increasing FcRn causes IgG to build up in the placental villous stroma, but FcγRIIb is the ultimate rate-limiting factor that determines whether IgG enters fetal circulation. High FcRn expression increases STB transcytosis efficiency and causes IgG to accumulate in the stroma “at the ready” for FcγRIIb-mediated transport, enabling FcγRIIb to transfer IgG at its maximum capacity. Conversely, when FcγRIIb expression is high, it is preferable to vaccinate later in pregnancy such that the peak maternal IgG post-immunization occurs when STBs express more FcRn to transport IgG into the stroma. We additionally found that the optimal t_vax_ is further influenced by the subclass of vaccine-induced IgG, a critical insight given that different pathogens are known to elicit different IgG subclasses (Fig 6F). These observations indicate that the role of FcγRIIb as the key rate-determining factor of IgG transfer is pertinent to the design of prenatal immunization strategies, such that increasing FcRn increases IgG transfer only when FcgRIIb is sufficiently expressed.

## Discussion

Placental antibody transfer is key to protect neonates against infection and to shape the trajectory of early-life immune development. Recently, maternal prenatal immunization has been employed to boost antigen-specific antibody transfer to the fetus, providing targeted immunity to the newborn. Current prenatal vaccine regimens are applied at the population level, but variation in gestational length, placental size and function, and maternal immune response presents an opportunity for patient-specific vaccine approaches. To this end, we developed a computational model to elucidate the features regulating maternal-fetal IgG transport and to develop novel vaccine strategies for precision prenatal immunization.

Our modeling approach enabled longitudinal probing of placental antibody transfer dynamics, shedding light on an otherwise black-boxed system. In contrast to prior studies attempting to uncover mechanisms of selective antibody transfer through a variety of *in vitro*, *ex vivo*, and *in vivo* approaches, our computational approach recapitulates this dynamic process as it occurs in humans. Our dynamic model predicted subclass-specific IgG transfer ratios that agree with the trend most commonly reported in the literature: IgG1 > IgG3 > IgG4 > IgG2. While the hierarchy was preserved, the model overestimated IgG3 transfer while underestimating IgG2 and IgG4 transfer. These subtle discrepancies may point to the involvement of other FcγRs such as FcγRIIIa (12,44), Hofbauer cells (7,9,16,20,45), or avidity effects that might arise from multivalent IgG-antigen complexes in fine-tuning antibody transfer dynamics (16,30). Notably, we disentangled the contributions of FcRn and FcγRIIb to EC transcytosis, revealing that in a baseline average placenta FcγRIIb drives IgG transcytosis in ECs and may play a key role in dynamic regulation of IgG transfer across gestation. Together, these observations elucidate the potential role of non-canonical FcγRs driving antibody transfer dynamics, though the role of FcγRIIb must be validated mechanistically *in vitro* to bolster our model-driven predictions.

Another key insight emerging from this analysis is the contribution of FcRs to transfer selectivity for particular IgG subclasses. In particular, subclass-specific IgG transfer is a function of FcR expression level, maternal IgG subclass abundance, and Fc-FcR interaction affinity. Thus, the subclass transfer hierarchy of IgG1 > IgG3 > IgG4 > IgG2 can be explained by each subclass’ relative abundance and affinity for FcRn and FcγRIIb. Not only that, but inter-patient variability in subclass abundances, FcR affinity arising from differential IgG glycosylation, and placental FcR expression can explain discrepancies in this subclass transfer hierarchy across individuals and patient cohorts.

The findings of this study have broad implications for prenatal vaccination, both at the population level and as a basis for patient-specific vaccine approaches. First, the model predicted that the optimal pertussis immunization time for an average patient is during the second trimester for term and preterm neonates alike, highlighting an opportunity to amend currently enforced vaccine regimens recommending Tdap immunization during the late second or early third trimester. From a precision medicine standpoint, recent studies suggest that careful modulation of vaccine parameters including the nature of the target antigen, conjugate, number of doses, route of entry, and choice of adjuvant can fine-tune the pool of maternal vaccine-induced antibodies and the anti-infection capabilities of the IgG transferred to the newborn. For example, protein antigens tend to induce an IgG1 or IgG3 response, while polysaccharide antigens tend to induce an IgG2 response (46); more recent studies suggest that the N-glycan profile of vaccine-induced IgG can be modulated by adjuvant, providing a means to fine-tune Fc receptor binding potential (47,48). The model-driven finding that IgG competes for placental transfer contingent upon subclass abundance, FcR expression, and Fc-FcR affinity provides a basis for improving vaccine formulations and dosing strategies to optimize vaccine-induced IgG transfer efficiency. Moreover, our model captures subclass-selectivity and subclass competition in placental transfer and is thus able to quantitatively assess the effect of a perturbed maternal pool of antibodies due to various immunodeficiency disorders (49), autoimmune disorders and other disorders that modify the maternal pool of antibodies and vaccine response. As such, our model has immense potential for devising vaccines for mothers with specific immunodeficiency disorders and in devising vaccine strategies aimed at optimizing user-defined selective set of antibodies in contrast with our objective of maximizing antigen-specific antibody titers in this study.

While many intriguing insights emerged from this model-driven investigation, there are a few limitations to consider. First, the current model relies on the assumption that all IgG is monovalent with a uniform glycan profile. This is an oversimplification as IgG glycosylation undergoes a dynamic shift during pregnancy (50), which can affect the Fc receptor-mediated transfer (12). Furthermore, in addition to monomeric IgG, IgG can potentially be transferred as antigen-antibody complexes, which is currently understudied and is not incorporated in our current model (16,30). Second, there is a stark lack of data surrounding human placental development and dynamic fetal IgG levels throughout gestation; many model parameters were unavailable in the literature, necessitating *in silico* parameter optimization. Consequently, we assumed several model components to be constant, including maternal, fetal, and placental volumes and maternal IgG levels. In reality, these features undergo rapid remodeling and growth across gestation and potentially fine-tune antibody transfer dynamics. Future iterations of the model may benefit from more physiologically accurate dynamics of the developing maternal-fetal interface. To facilitate such a model, more studies detailing the dynamics of placental growth—especially as it pertains to vascularization and trophoblast differentiation to form STB layers—and of FcR expression at the protein level are warranted. To infer the role of these dynamic placental changes in antibody transfer, placental samples should be analyzed in conjunction with antibody profiling in matched maternal and umbilical cord serum.

Despite its limitations, a key strength of our computational approach is the ability to simulate placental transport dynamics which are prohibitively challenging to study *in vivo*. Using tightly integrated modeling and experimental methods, we probed placental transport across time and size scales, enabling a more holistic view of the system compared to previous studies in the field. Traditionally, placental IgG transport efficiency is benchmarked by a simple ratio of maternal to umbilical cord IgG titer at birth, in part due to its ease of measurement and interpretability. In addition to masking the nuances of maternal pool of antibodies, this metric insufficiently describes the highly dynamic and cumulative process of antibody transfer and is thus not capable of describing: (1) important concepts such as placental FcR limitation and antibody subclass competition, (2) the interplay between the maternal pool of antibodies and the vaccine-induced maternal antibodies, and (3) the role of the complex placental functions that rapidly evolve throughout gestation, among other reasons. Our mathematical model challenges this metric as a true measure of “efficiency”. With the advent and accessibility of state-of-the-art high-throughput measurements, it is increasingly feasible to probe placental function and define one or more quantitative measures of transfer efficiency that are conditioned on the maternal pool of antibodies and are linked with distinct model parameters that account for placental size, perfusion, and Fc receptor expression.

This study represents a major thrust forward in the field of maternal-fetal immunology and paves the way towards next generation vaccination programs to fine-tune neonatal immunity against a broad range of pathogens. As current vaccination regimens are continually revised and several novel vaccines are currently in clinical trials, now an opportune time to increase the prevalence of computational systems biology approaches in maternal-fetal medicine to improve outcomes for vulnerable newborns.

## Methods

### Mechanistic model of placental IgG transport

An ordinary differential equation (ODE) model was formulated to simulate mass transport of IgG1, IgG2, IgG3, and IgG4 across the maternal-fetal interface (Appendix S1). A schematic of the model is given in Figure 1A. Fundamental model assumptions are as follows: (i) all processes obey the law of mass action; (ii) IgG binds FcRn within STB endosomes at an acidified pH and FcγRIIb at the EC surface; (iii) binding with either receptor initiates transcytosis; (iv) the effects of FcRn-mediated IgG recycling to the apical STB surface are negligible; (v) unbound IgG in STB endosomes undergoes lysosomal degradation; (vi) all blood volumes, IgG subclass concentrations in maternal blood, and IgG-FcR affinities remain constant throughout gestation; (vii) rate parameters (k_up_, k_trans_, and k_deg_) are equal in STB and EC layers and across IgG subclasses; (viii) stromal cells (e.g., Hofbauer cells, fibroblasts) do not interact with IgG or affect its transfer; (ix) the effect of Fab-antigen interactions is neglected such that all IgG is monovalent; (x) FcR expression increases parabolically across gestation (51) informed by FcRn expression trends in rat placenta; (xi) STBs exclusively express FcRn and ECs exclusively express FcγRIIb. All model development and corresponding *in silico* experiments were performed in MATLAB R2022a (Mathworks).

### Parameter estimation

Model parameters were derived from literature when possible, including K_D_ parameters, maternal IgG concentrations, and all compartment volumes (29,31,52,53). Remaining parameter ranges were determined using CaliPro, a flexible optimization software implemented in MATLAB and described by Joslyn et al (28). A key strength of CaliPro is its ability to home in on a biologically relevant range of parameters that recapitulate the system dynamics rather than minimizing a simple cost function. Briefly, we implemented CaliPro using Latin Hypercube Sampling (LHS) from a physiologically plausible range of parameter values as determined by benchmarking literature studies. We defined the pass criteria as model simulations within the upper standard deviation of the data set at the first time point, and are within 1.5 times the standard deviation at the final time point. Our termination criteria was set to 95% of model runs meeting the pass criteria.

### Global sensitivity analysis

Global sensitivity analysis was performed using an orthogonalized partial least squares regression (OPLSR) framework, as described by Surve et al (34). 500 perturbed parameter sets were generated by LHS from a uniform distribution spanning the range for each parameter obtained from CaliPro. An OPLSR model was constructed using the matrix of 500 permuted parameter sets to predict the total concentration of IgG in the fetus at 40 weeks gestation. A second OPLSR model was constructed in the same manner to predict entropy among IgG subclasses in the fetus at 40 weeks gestation, defined as:

$$P_{IgGi}=\frac{IgG_{i}}{\sum_{i=1}^{4}\;IgG_{i}}$$

$$E=-\sum_{i=1}^{4}P_{IgGi}\log\left(P_{IgGi}\right)$$

Where E is entropy and $P_{IgGi}$ is the proportion of each subclass in the fetal blood. Model orthogonalization was carried out based on the published method (54). OPLSR model significance was determined empirically by comparing its predictive power (Q^2^) against randomly permuted models.

### HUVEC transcytosis assay

#### Antibody preparation

Purified human polyclonal IgG1 and IgG4 (Abcam, AB90283, AB183266) were dialyzed in 1x PBS using Slide-A-Lyzer mini dialysis units with a 10K molecular weight cutoff (Invitrogen, 88404) to remove sodium azide. Dialysis units were incubated at 4°C for 2 hours, then dialysis buffer was changed and incubated again at 4°C for 2 hours. Protein was removed from the dialysis unit using a micropipette and quantified using a NanoDrop Microvolume Spectrophotometer (Thermo Fisher).

#### Cell culture

6.5 mm diameter Transwell inserts (Corning, CLS3422) coated in 1.4% gelatin were seeded with primary human umbilical vein endothelial cells (HUVEC) (Promocell C-12200, kindly provided by Dan Gioeli at UVA) at a density of 1500 cells/mm^2^ (50,000 cells/insert). Cells were cultured in complete endothelial cell growth media (Promocell, C-22110) and incubated at 37°C and 5% CO_2_. After 24 hours, the cells were washed in sterile PBS and the media was changed.

#### Transcytosis assay

After 48 hours, the cells were washed in sterile PBS and media containing purified human IgG was added to the apical chamber. Cells were incubated at 37°C and 5% CO_2_ for 120 minutes. IgG1 and IgG4 in the basolateral media were quantified using human enzyme-linked immunosorbent assay (ELISA) IgG1 and IgG4 quantification kits (Invitrogen, EHIGG1 and BMS2095) according to the manufacturer’s protocol. All conditions were run in triplicate and ELISA measurements were run in duplicate. A 5-parameter logistic standard curve was generated for each assay. Absorbance was read at 450 nm with a correction of 590 nm using an Optima microplate reader within 1 hour of completing the assay. Statistical significance was determined with a one-tailed two-sample t-test comparing each sample containing IgG1 and IgG4 to the sample with IgG4 but without IgG1.

To confirm monolayer formation, membrane permeability was quantified for each assay by addition of FITC-dextran (4000 MW, Sigma Aldrich 46944) (0.5 mg/ml) to the apical chamber of each insert. FITC-dextran added to inserts without gelatin coating or HUVEC served as a negative control. The basolateral media was sampled after 120 minutes, and fluorescence was read using the Optima microplate reader. Monolayer permeability (%) was defined as:

$$Permeability\;=\frac{[basolat.FITC-\;dextran{]}_{HUVEC}}{[basolat.FITC-\;dextran{]}_{blank}}\times 100$$

Samples with FITC-dextran permeability greater than 3 standard deviations from the mean were excluded based on faulty monolayer formation (S5 Fig).

### Mechanistic model of HUVEC transcytosis *in vitro*

To simulate the dynamics of IgG1 and IgG4 receptor-mediated transcytosis in HUVEC, a kinetic-dynamic model of FcRn-IgG binding and transcytosis in endothelial cells was formulated:

(1) $$\frac{dIgG_{1}}{dt}=-k_{on,1}\left[IgG_{1}\right][FcRn]+k_{off,1}\left[C_{1}\right]$$

(2) $$\frac{dIgG_{4}}{dt}=-k_{on,4}\left[IgG_{4}\right][FcRn]+k_{off,4}\left[C_{4}\right]$$

(3) $$\frac{dFcRn}{dt}=-[FcRn]\left(k_{on,1}\left[IgG_{1}\right]+k_{on,4}\left[IgG_{4}\right]\right)+\left(k_{off,1}+k_{T}\right)\left[C_{1}\right]+\left(k_{off,4}+k_{T}\right)\left[C_{4}\right]$$

(4) $$\frac{dC_{1}}{dt}=k_{on,1}\left[IgG_{1}\right][FcRn]-\left(k_{off,1}+k_{T}\right)\left[C_{1}\right]\;$$

(5) $$\frac{dC_{4}}{dt}=k_{on,4}\left[IgG_{4}\right][FcRn]-\left(k_{off,4}+k_{T}\right)\left[C_{4}\right]$$

(6) $$\frac{dIgG_{T,1}}{dt}=k_{T}\left[C_{1}\right]$$

(7) $$\frac{dIgG_{T,4}}{dt}=k_{T}\left[C_{4}\right]$$

Where [IgG1] and [IgG4] are apical concentrations of IgG1 and IgG4, [FcRn] is the concentration of FcRn expression in HUVEC, $\left[C_{1}\right]$ and $\left[C_{4}\right]$ are the concentration of bound IgG-FcRn complexes, $k_{T}$ is the rate of transcytosis, $k_{on}$ is the forward rate of reaction, and $k_{\text{off}\;}$ is the reverse rate of reaction (Table 2). The model parameters were optimized by fitting to dynamic data of IgG4 transcytosis in HUVECs collected at 15, 30, 60, and 120 minutes ($\left[\mathrm{lgG}4_{\text{apical}}\right]=0.5$ and 0.125 mg/ml) (S5 Fig). To simplify this model and reveal the effect of subclass-subclass competition in Fc receptor-mediated transcytosis, we derived the closed form of equation (5). We assumed that IgG1 and IgG4 are present in excess of FcRn, the system is at quasi-steady state such that

(8) $$\frac{dC_{1}}{dt}=\frac{dC_{4}}{dt}=0$$

Where C is the concentration of IgG-FcRn complexes. Under these assumptions, equations (4) and (5) can be rearranged as

(9) $$\frac{k_{on,1}\left[IgG_{1}\right][FcRn]}{k_{on,1}\left[IgG_{1}\right][FcRn]\left(k_{off,1}\;+\;k_{T}\right)}=\left[C_{1}\right]$$

(10) $$\begin{array}{r} \frac{k_{on,4}\left[IgG_{4}\right]\left[F_{CRn}\right]}{k_{on,4}\left[IgG_{4}\right]\left[F_{CRn}\right]\left(k_{off,4}\;+\;k_{T}\right)}=\left[C_{4}\right] \end{array}$$

The assumption is then made that the total concentration of receptor does not change such that

(11) $$\;[FcRn]=\left[FcRn_{0}\right]-\left[C_{1}\right]-\left[C_{4}\right]$$

Substituting (11) into (10):

(12) $$\frac{k_{on,4}\left[IgG_{4}\right]\left(\left[FcRn_{0}\right]\;-\;\left[C_{1}\right]\;-\;\left[C_{4}\right]\right)}{k_{on,4}\left[IgG_{4}\right][FcRn]\left(k_{off,4}\;+\;k_{T}\right)}=\left[C_{4}\right]$$

And (9) into (12):

(13) $$\frac{k_{on,4}\left[IgG_{4}\right](\left[FcRn_{0}\right]-\frac{k_{on,1}\left[IgG_{1}\right][FcRn]}{k_{on,1}\left[IgG_{1}\right][FcRn]\left(k_{off,1}\;+\;k_{T}\right)}-\left[C_{4}\right])}{k_{on,4}\left[gG_{4}\right][FcRn]\left(k_{off,4}\;+\;k_{T}\right)}=\left[C_{4}\right]$$

Solving (13) for $\left[C_{4}\right]$ yields the closed-form solution for IgG4-FcRn complex formation (55):

(14) $$C_{4}=\frac{FcRn_{0}\left[IgG_{4}\right]}{K_{D,4}(1\;+\;\frac{\left[IgG_{4}\right]}{K_{D,4}}\;+\;\frac{\left[IgG_{1}\right]}{K_{D,1}})}$$

Which quantifies the effect of competition between two IgG subclasses undergoing transcytosis in the presence of one Fc receptor.

### Pertussis immunization simulations

A model of plasma cell activation and IgG secretion in response to antigen stimulus was adapted from a previous study by White et al in the context of malaria (38). Parameters were tuned to fit previously published dynamic data of the maternal IgG response to Tdap booster vaccination (39) (Table 3). We assumed several parameters which depend on the host environment—namely, systemic cytokine concentrations and availability of FcRn for IgG recycling and half-life extension—do not depend on antigen-specificity and thus are conserved between malaria and pertussis contexts (56,57). Such parameters include the proportion of long-lived antibody secreting cells (L_ASC_) to short-lived antibody secreting cells (S_ASC_), the lifespan of L_ASC_ and S_ASC_, and the antibody decay rate and were preserved from White et al (38). On the other hand, parameters which depend on the plasma cell niche and context of the specific antigenic challenge (e.g., rates of plasma cell proliferation, antibody secretion, and the antigen decay rate) were optimized to fit titers of anti-pertussis toxin IgG (α-PT IgG) in women of childbearing age following a booster Tdap vaccination (39) (Table 3). Other important model assumptions include: (i) IgG has a half-life of 31 days in the fetus; (ii) the dynamics of B cell activation—including antigen presentation by follicular dendritic cells and cytokine secretion by T cells—and the contribution of memory B cells are neglected; (iii) mothers have been previously exposed to pertussis toxin (i.e., this is a booster vaccine); (iv) all mothers have the same dynamic response to immunization; (v) Immunization resulted in equal parts IgG1, IgG2, IgG3, and IgG4 with uniform glycosylation profile (i.e., glycosylation does not affect FcRn nor FcγRIIb binding) and immunoglobulin isotype switching was neglected; (vi) L_ASC_s and S_ASC_s secrete IgG with identical subclass and glycosylation profile; (vii) the peak antibody titer between 14 and 28 days post-immunization; (viii) the IgG response to pertussis is the same in pregnant and non-pregnant individuals(58). Maternal immunization was simulated by stimulating the maternal compartment with antigen at a time t_vax_ (given in weeks gestational age) to induce an antibody response, which directly fed into the maternal compartment of the placental IgG transport model. To determine the optimal t_vax_ corresponding to maximum pertussis-toxin IgG transfer to the fetus, iterative simulations were performed over a range of t_vax_ spanning 10–38 weeks gestational age. Sensitivity of the optimal t_vax_ was determined by parameter perturbation and subsequent PLSR model construction, as described above. When perturbing the IgG subclass composition induced by immunization, it was assumed that that vaccine induced solely IgG1, IgG2, IgG3, or IgG4.

## Supplementary Material

Supplement 1

## Figures and Tables

**Table 1.** List of optimized model parameters.

Parameter	Value	Units	Interpretation	Source
k_up_	0.0567–0.0938	L/week	Uptake rate into cells	Optimized
k_t_	0.0684–0.1071	L/week	Transcytosis rate	Optimized
k_deg_	4.1635–5.0418	L/week	Lysosomal degradation rate	Optimized
FcRn	6.54–6.794e-6	M	STB FcRn expression	Optimized
FcγRIIb	3.096–3.607e-6	M	EC FcγRIIb expression	Optimized
IgG1_0_	3.78e-5	M	Maternal IgG1 concentration	(29)
IgG2_0_	1.81e-5	M	Maternal IgG2 concentration	(29)
IgG3_0_	2.35e-6	M	Maternal IgG3 concentration	(29)
IgG4_0_	2.27e-6	M	Maternal IgG4 concentration	(29)
K_D_, FcRn-IgG1	1.25e-8	M	FcRn and IgG1 dissociation constant	(31)
K_D_, FcRn-IgG2	2e-8	M	FcRn and IgG2 dissociation constant	(31)
K_D_, FcRn-IgG3	3.3e-8	M	FcRn and IgG3 dissociation constant	(31)
K_D_, FcRn-IgG4	5e-8	M	FcRn and IgG4 dissociation constant	(31)
K_D_, FcγRIIb-IgGI	3.1e-6	M	FcγRIIb and IgG1 dissociation constant	(31)
K_D_, FcγRIIb-IgG2	6.8e-6	M	FcγRIIb and IgG2 dissociation constant	(31)
K_D_, FcγRIIb-IgG3	1.3e-6	M	FcγRIIb and IgG3 dissociation constant	(31)
K_D_, FcγRIIb-IgG4	1.7e-6	M	FcγRIIb and IgG4 dissociation constant	(31)
V_M_	6.425–6.7255	L	Maternal blood volume	Optimized
V_STB_	0.0901–0.0924	L	Total STB endosomal volume	Optimized
V_STR_	0.0752–0.083	L	Stromal volume	Optimized
V_EC_	0.0235–0.0275	L	Total EC endosomal volume	Optimized
V_F_	0.1534–0.1633	L	Fetal blood volume	Optimized
δ_Ab_	0.02	L/week	IgG decay rate in the fetus	(32)

**Table 2.** Optimized HUVEC transcytosis model parameters.

Parameter	Value	Units	Interpretation
FcRn	2200	nM	HUVEC FcRn expression level
K_deg_	0.01	1/minute	Lysosomal degradation rate
K_t_	2.5 × 10^−5^	1/minute	Transcytosis rate

**Table 3.** Pertussis immunization 324 model parameters.

Parameter	Value	Units	Interpretation	Source
k_ASC_	0.6	1 / 10^6^ PBMCs / day / Ag	Rate of plasma cell generation	Optimized
k_Ab_	0.0475	mg / ml * (10^6^ PBMCs) / days	Rate of antibody production by plasma cells	Optimized
Ag_0_	100	mg/ml	Initial dose of antigen	Optimized
ρ	96	%	Proportion of short-lived ASCs (S_ASC_)	(38)
δ_Ag_	0.15	1 / day	Antigen decay rate	(42)
δ_Ab_	0.033	1 / day	Antibody decay rate	(38)
δ_S-ASC_	0.578	1 / day	Short-lived antibody-secreting cell (S_ASC_) decay rate	(38)
δ_L-ASC_	6.6 × 10^−4^	1 / day	Long-lived antibody secreting cell (L_ASC_) decay rate	(38)
