# Supplementary material for: Quantitative mechanistic model reveals key determinants of placental IgG transfer and informs prenatal immunization strategies
Source: bioRxiv. 2023 Apr 19:2023.04.18.537285. Preprint. [Version 2] doi: 10.1101/2023.04.18.537285 (PMC10153162; doi:10.1101/2023.04.18.537285)
Supplement: Supplementary file 1 [file media-1.pdf]

Supporting information

Here we include Supplementary Figures S1-S5, which provide additional evidence, further exploration of and insights into the model and data. Also included here are the placental IgG transfer model equations as well as the maternal Tdap vaccination model equations, which was integrated with this placental model.

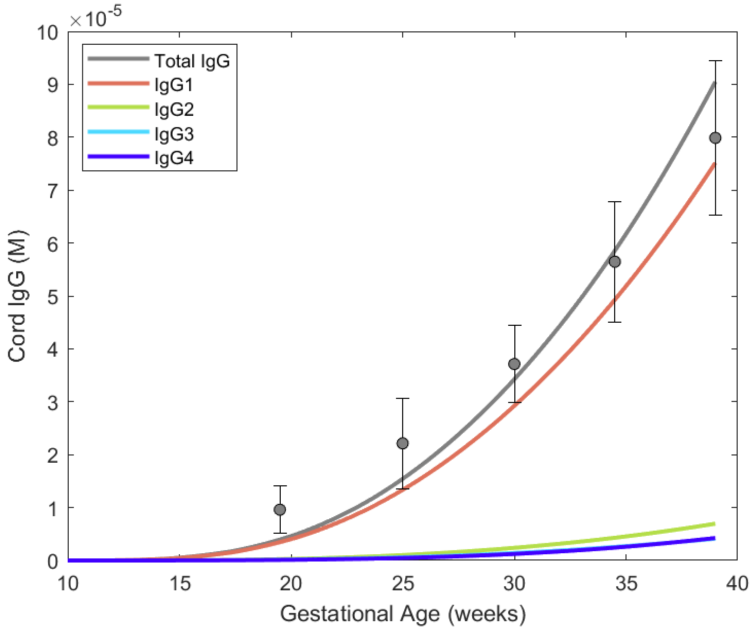

Figure S1. The mechanistic model recapitulates bulk IgG transfer. Bulk IgG in umbilical cord blood measured using cordocentesis in a cohort spanning gestational ages is shown as the cohort mean and standard deviation in grey circles. Simulated total fetal IgG and IgG subclasses across gestation are overlaid as solid curve. The curve for IgG3 is covered by the curve for IgG4.

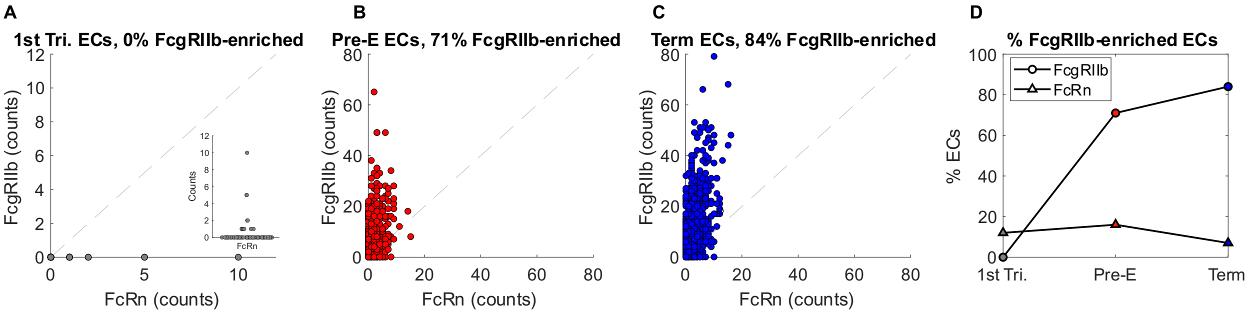

Figure S2. Preferential expression of FCGR2B over FCGRT by endothelial cells increases across gestation. (A-C) The scatter plots show the expression of FCGR2B and FCGRT in placental ECs in scRNA-seq data sets from three gestational time points: (A) first trimester, (B) early third trimester, and (C) late third trimester. Each dot corresponds to a single cell. Line y = x is indicated on each panel. In panel (A), a violin plot of FcRn expression is included to more clearly demonstrate the abundance of cells at the origin. (D) The trend of FcRn and FcgRIIb expression is shown across gestation as the percentage of ECs expressing FcgRIIb (circles) and FcRn (triangles) at each time point.

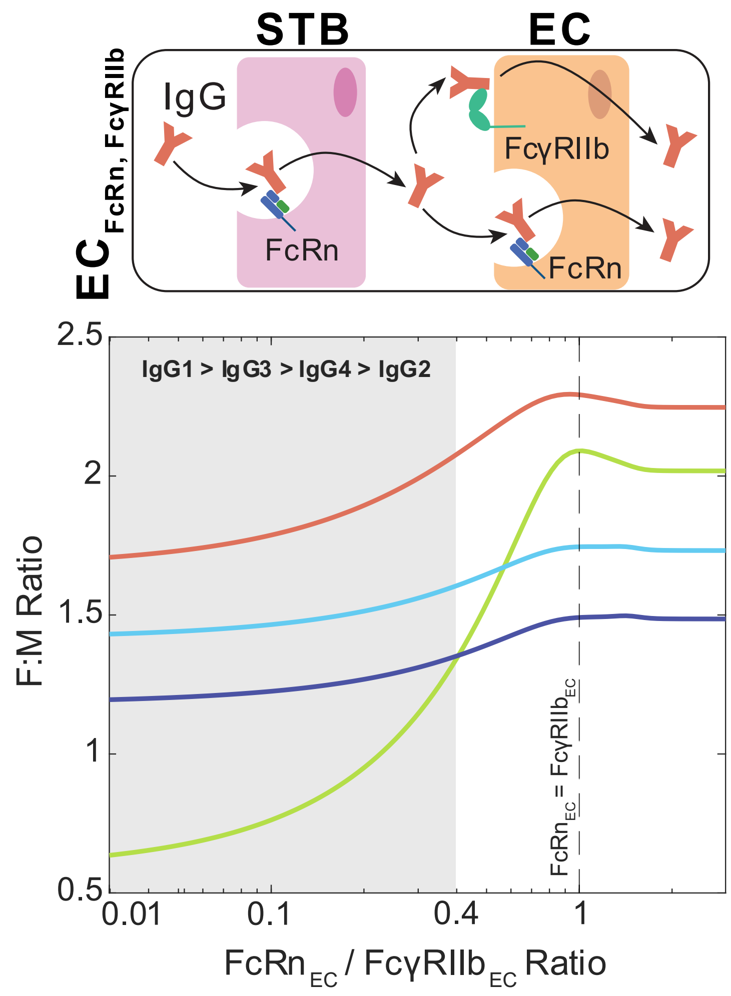

Figure S3. Subclass-specific transfer occurs with co-expression of FcRn and FcgRIIb when FcgRIIb > FcRn. (A) Model-simulated IgG subclass transfer ratios as a function of the ratio of FcRn to FcγRIIb in placental ECs expressing both FcRn and FcγRIIb ($EC_{FcRn,FcγRIIb}$). The shaded region shows the regime of FcRn expression where the subclass transfer hierarchy IgG1 > IgG3 > IgG4 > IgG2 is preserved. The dashed line represents equal FcRn and FcγRIIb expression.

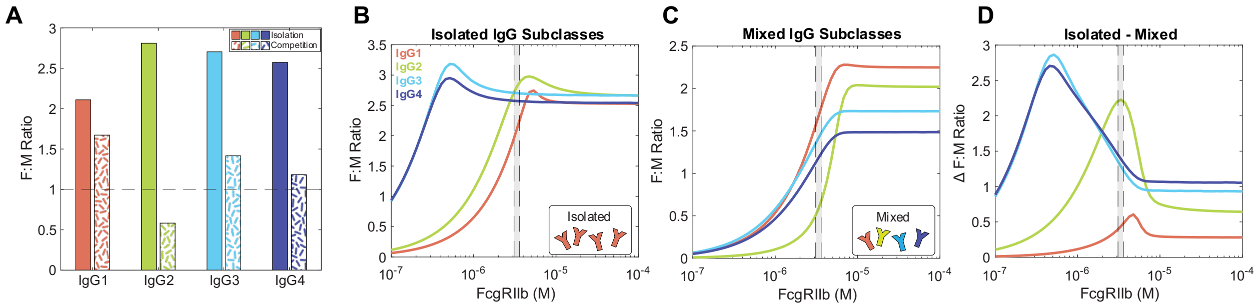

Figure S4. IgG subclasses compete for FcgRIIb-mediated transcytosis in the placental mechanistic model. **(A)** The bar plot represents the F:M ratio at parturition for a term pregnancy from independent simulations in the compartmental placental model with each subclass isolated (solid fill) or mixed with other IgG subclasses ("Competition", patterned fill). **(B-C)** The F:M ratio at parturition for a term pregnancy is shown for each subclass in isolation **(B)** or mixed with other subclasses **(C)** as in **(A)** over a range of FcgRIIb expression. **(D)** The difference (delta) in F:M ratio at parturition for a term pregnancy between isolation and competition conditions is shown over a range of FcgRIIb expression. In **(B-D),** the optimized range of FcgRIIb expression is indicated by the shaded region.

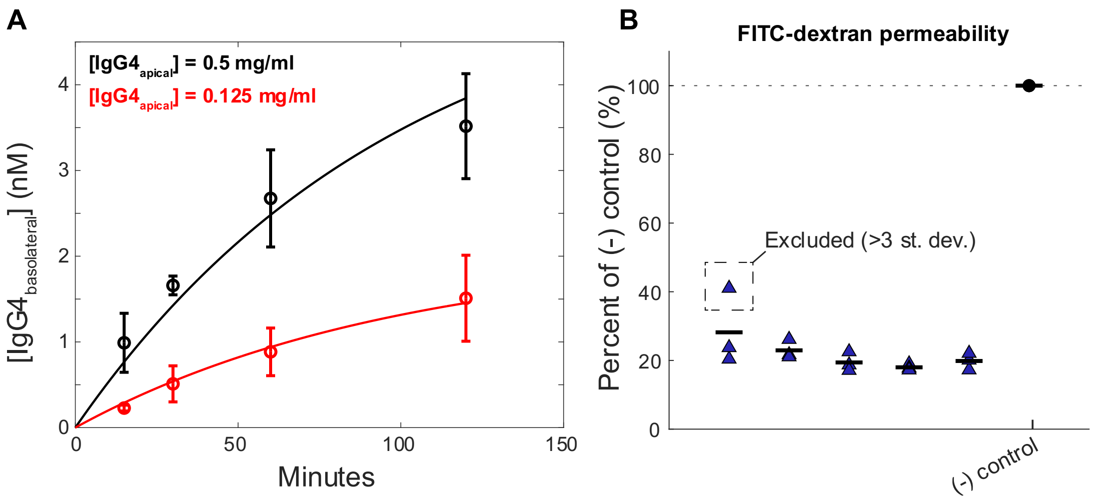

Figure S5. Transwell simulation model parameters were fit to longitudinal *in vitro* data. (A) Longitudinal dynamic data from IgG transcytosis measured *in vitro* in a HUVEC model are shown as the mean and standard deviation of 3 replicates. The overlaid curves show corresponding model simulations described in Methods. (B) The FITC-dextran permeability measurements demonstrates the criteria for rejecting a data point on the basis of faulty monolayer formation.

Placental IgG transfer model equations

Equations 1-4 represent IgG concentration in the maternal plasma compartment:

$$\frac{dIgG1_M}{dt} = 0 \quad (1)$$

$$\frac{dIgG2_M}{dt} = 0 \quad (2)$$

$$\frac{dIgG3_M}{dt} = 0 \quad (3)$$

$$\frac{dIgG4_M}{dt} = 0 \quad (4)$$

Equations 5-12 represent IgG in STB endosomes, either as monovalent IgG (5-8) or IgG in complex with FcRn in STB endosomes:

$$\frac{dIgG1_{STB}}{dt} = [k_{off,1}C_{IgG1,FcRn} + k_{up}IgG1_M - k_{on,1}IgG1_{STB}FcRn_{STB} - k_{deg}IgG1_{STB}]\frac{1}{v_{STB}} \quad (5)$$

$$\frac{dIgG2_{STB}}{dt} = [k_{off,2}C_{IgG2,FcRn} + k_{up}IgG2_M - k_{on,2}IgG2_{STB}FcRn_{STB} - k_{deg}IgG2_{STB}]\frac{1}{v_{STB}} \quad (6)$$

$$\frac{dIgG3_{STB}}{dt} = [k_{off,3}C_{IgG3,FcRn} + k_{up}IgG3_M - k_{on,3}IgG3_{STB}FcRn_{STB} - k_{deg}IgG3_{STB}]\frac{1}{v_{STB}} \quad (7)$$

$$\frac{dIgG4_{STB}}{dt} = [k_{off,4}C_{IgG4,FcRn} + k_{up}IgG4_M - k_{on,4}IgG4_{STB}FcRn_{STB} - k_{deg}IgG4_{STB}]\frac{1}{v_{STB}} \quad (8)$$

$$\frac{dC_{IgG1,FcRn}}{dt} = [-k_{off,1}C_{IgG1,FcRn} + k_{on,1}IgG1_{STB}FcRn_{STB} - k_tC_{IgG1,FcRn}]\frac{1}{v_{STB}} \quad (9)$$

$$\frac{dC_{IgG2,FcRn}}{dt} = \left[-k_{off,2}C_{IgG2,FcRn} + k_{on,2}IgG2_{STB}FcRn_{STB} - k_tC_{IgG2,FcRn}\right]\frac{1}{v_{STB}} \quad (10)$$

$$\frac{dC_{IgG3,FcRn}}{dt} = \left[-k_{off,3}C_{IgG3,FcRn} + k_{on,3}IgG3_{STB}FcRn_{STB} - k_tC_{IgG3,FcRn}\right]\frac{1}{v_{STB}} \quad (11)$$

$$\frac{dC_{IgG4,FcRn}}{dt} = \left[-k_{off,4}C_{IgG4,FcRn} + k_{on,4}IgG4_{STB}FcRn_{STB} - k_tC_{IgG4,FcRn}\right]\frac{1}{v_{STB}} \quad (12)$$

Equation 13 represents the concentration of FcRn expressed within STB endosomes:

$$\frac{dFcRn}{dt} = [k_{off,1}C_{IgG1,FcRn} + k_{off,2}C_{IgG2,FcRn} + k_{off,3}C_{IgG3,FcRn} + k_{off,4}C_{IgG4,FcRn} - \left(k_{on,1}IgG1_{STB} + k_{on,2}IgG2_{STB} + k_{on,3}IgG3_{STB} + k_{on,4}IgG4_{STB}\right)FcRn_{STB} + k_t\left(C_{IgG1,FcRn} + C_{IgG2,FcRn} + C_{IgG3,FcRn} + C_{IgG4,FcRn}\right) + \Delta FcRn]\frac{1}{v_{STB}} \quad (13)$$

Where ΔFcRn is the derivative of the second order polynomial curve fit representing increasing FcRn concentration across gestation:

$$FcRn = at^2 + bt + c \quad (14)$$

$$\Delta\, FcRn = 2at + b \quad (15)$$

Where a, b, and c are parameters determined for the FcRn concentration in a given simulation. FcRn binding shuttles IgG through STBs and into the intervillous stroma. IgG concentration in the stroma is represented by equations 16-19:

$$\frac{dIgG1_{STR}}{dt} = [k_{off,1}C_{IgG1,Fc\gamma RIIb} + k_tC_{IgG1,FcRn} - k_{on,1}IgG1_{STR}Fc\gamma RIIb_{STR}]\frac{1}{v_{STR}} \quad (16)$$

$$\frac{dIgG2_{STR}}{dt} = [k_{off,2}C_{IgG2,Fc\gamma RIIb} + k_tC_{IgG2,FcRn} - k_{on,2}IgG2_{STR}Fc\gamma RIIb_{STR}]\frac{1}{v_{STR}} \quad (17)$$

$$\frac{dIgG3_{STR}}{dt} = [k_{off,3}C_{IgG3,Fc\gamma RIIb} + k_tC_{IgG3,FcRn} - k_{on,3}IgG3_{STR}Fc\gamma RIIb_{STR}]\frac{1}{v_{STR}} \quad (18)$$

$$\frac{dIgG4_{STR}}{dt} = [k_{off,4}C_{IgG4,Fc\gamma RIIb} + k_tC_{IgG4,FcRn} - k_{on,4}IgG4_{STR}Fc\gamma RIIb_{STR}]\frac{1}{v_{STR}} \quad (19)$$

IgG then binds to FcγRIIb on the EC surface. The concentration of IgG subclasses and FcγRIIb in complex is given by equations 20-23:

$$\frac{dC_{IgG1,Fc\gamma RIIb}}{dt} = \left[-k_{off,1}C_{IgG1,Fc\gamma RIIb} + k_{on,1}IgG1_{STR}Fc\gamma RIIb_{EC} - k_{up}C_{IgG1,Fc\gamma RIIb}\right]\frac{1}{v_{STR}} \quad (20)$$

$$\frac{dC_{IgG2,Fc\gamma RIIb}}{dt} = \left[-k_{off,2}C_{IgG2,Fc\gamma RIIb} + k_{on,2}IgG2_{STR}Fc\gamma RIIb_{EC} - k_{up}C_{IgG2,Fc\gamma RIIb}\right]\frac{1}{v_{STR}} \quad (21)$$

$$\frac{dC_{IgG3,Fc\gamma RIIb}}{dt} = \left[-k_{off,3}C_{IgG3,Fc\gamma RIIb} + k_{on,3}IgG3_{STR}Fc\gamma RIIb_{EC} - k_{up}C_{IgG3,Fc\gamma RIIb}\right]\frac{1}{v_{STR}} \quad (22)$$

$$\frac{dC_{IgG4,Fc\gamma RIIb}}{dt} = \left[-k_{off,4}C_{IgG4,Fc\gamma RIIb} + k_{on,4}IgG4_{STR}Fc\gamma RIIb_{EC} - k_{up}C_{IgG4,Fc\gamma RIIb}\right]\frac{1}{v_{STR}} \quad (23)$$

And the concentration of free FcγRIIb is given by equation 24:

$$\frac{dFc\gamma RIIb}{dt} = [k_{off,1}C_{IgG1,Fc\gamma RIIb} + k_{off,2}C_{IgG2,Fc\gamma RIIb} + k_{off,3}C_{IgG3,Fc\gamma RIIb} + k_{off,4}C_{IgG4,Fc\gamma RIIb} - \left(k_{on,1}IgG1_{STB} + k_{on,2}IgG2_{STB} + k_{on,3}IgG3_{STB} + k_{on,4}IgG4_{STB}\right)Fc\gamma RIIb_{EC} + k_t\left(C_{IgG1,Fc\gamma RIIb} + C_{IgG2,Fc\gamma RIIb} + C_{IgG3,Fc\gamma RIIb} + C_{IgG4,Fc\gamma RIIb}\right) + \Delta Fc\gamma RIIb]\frac{1}{v_{STR}} \quad (24)$$

Where Δ FcγRIIb is the derivative of the second order polynomial curve fit representing increasing FcγRIIb concentration across gestation:

$$Fc\gamma RIIb = at^2 + bt + c \quad (25)$$

$$\Delta\, Fc\gamma RIIb = 2at + b \quad (26)$$

Where a, b, and c are parameters determined for the FcγRIIb concentration in a given simulation. FcγRIIb binding results in IgG being taken up into EC endosomes where IgG dissociates from FcγRIIb and is trafficked across the ECs. The concentration of IgG within EC endosomes is given by equations 27-30:

$$\frac{dIgG1_{EC}}{dt} = [k_{up}C_{IgG1,Fc\gamma RIIb} - k_t IgG1_{EC}]\frac{1}{v_{EC}} \quad (27)$$

$$\frac{dIgG2_{EC}}{dt} = [k_{up}C_{IgG2,Fc\gamma RIIb} - k_t IgG2_{EC}]\frac{1}{v_{EC}} \quad (28)$$

$$\frac{dIgG3_{EC}}{dt} = \left[k_{up}C_{IgG3,Fc\gamma RIIb} - k_t IgG3_{EC}\right]\frac{1}{v_{EC}} \quad (29)$$

$$\frac{dIgG4_{EC}}{dt} = \left[k_{up}C_{IgG4,Fc\gamma RIIb} - k_t IgG4_{EC}\right]\frac{1}{v_{EC}} \quad (30)$$

The IgG concentration in the fetus is then represented by equations 31-34:

$$\frac{dIgG1_F}{dt} = [k_t IgG1_{EC} - \delta_{Ab}]\frac{1}{v_F} \quad (31)$$

$$\frac{dIgG2_F}{dt} = [k_t IgG2_{EC} - \delta_{Ab}] \frac{1}{v_F} \quad (32)$$

$$\frac{dIgG3_F}{dt} = [k_t IgG3_{EC} - \delta_{Ab}] \frac{1}{v_F} \quad (33)$$

$$\frac{dIgG4_F}{dt} = [k_t IgG4_{EC} - \delta_{Ab}] \frac{1}{v_F} \quad (34)$$

Tdap immunization model equations

Equations 35-38 represent the naïve B cell response to maternal immunization against pertussis toxin. The concentration of antigen injected intramuscularly in the mother is given in equation 35:

$$\frac{dAg}{dt} = \begin{cases} t < t_{vax}, & 0 \\ t \geq t_{vax}, & [-\delta_{Ag} Ag] \frac{1}{v_M} \end{cases} \quad (35)$$

And is equal to 0 prior to t_{vax}. Equation 36 and 37 represent the concentration of short-lived antibody secreting cells (S_{ASC}) and long-lived antibody secreting cells (L_{ASC}) specific to pertussis toxin following immunization:

$$\frac{dS_{ASC}}{dt} = \begin{cases} t < t_{vax}, & 0 \\ t \geq t_{vax}, & [\rho k_{ASC} - \delta_{S-ASC} S_{ASC}] \frac{1}{v_M} \end{cases} \quad (36)$$

$$\frac{dL_{ASC}}{dt} = \begin{cases} t < t_{vax}, & 0 \\ t \geq t_{vax}, & [(1-\rho) k_{ASC} - \delta_{L-ASC} L_{ASC}] \frac{1}{v_M} \end{cases} \quad (37)$$

The concentration of IgG in the mother specific to pertussis toxin is represented by equation 38:

$$\frac{dIgG_{M,Ag}}{dt} = \begin{cases} t < t_{vax}, & 0 \\ t \geq t_{vax}, & [k_{IgG}(S_{ASC} + L_{ASC}) - \delta_{IgG} IgG_{M,Ag}] \frac{1}{v_M} \end{cases} \quad (38)$$

To perform pertussis toxin immunization simulations, equations 35-38 are augmented into the model described by equations 1-34. New equations similar to 5-12, 16-23, and 27-34 are added to this set to represent anti-pertussis toxin IgG1, IgG2, IgG3, and IgG4 in STB endosomes, the

stroma, EC endosomes, and the fetus. Rate-limiting equations 13 and 24 are adjusted to account for competition between bulk IgG1-4 and anti-pertussis toxin IgG1-4 for FcRn and FcγRIIb binding, respectively:

$$\frac{dFcRn}{dt} = \Big[k_{off,1}(C_{IgG1,FcRn} + C_{IgG1,FcRn}^{\alpha-PT}) + k_{off,2}(C_{IgG2,FcRn} + C_{IgG2,FcRn}^{\alpha-PT}) + k_{off,3}(C_{IgG3,FcRn} + C_{IgG3,FcRn}^{\alpha-PT}) + k_{off,4}(C_{IgG4,FcRn} + C_{IgG4,FcRn}^{\alpha-PT}) - \big(k_{on,1}(IgG1_{STB} + IgG1_{STB}^{\alpha-PT}) + k_{on,2}(IgG2_{STB} + IgG2_{STB}^{\alpha-PT}) + k_{on,3}(IgG3_{STB} + IgG3_{STB}^{\alpha-PT}) + k_{on,4}(IgG4_{STB} + IgG4_{STB}^{\alpha-PT})\big)FcRn_{STB} + k_t\big(C_{IgG1,FcRn} + C_{IgG2,FcRn} + C_{IgG3,FcRn} + C_{IgG4,FcRn} + C_{IgG1,FcRn}^{\alpha-PT} + C_{IgG2,FcRn}^{\alpha-PT} + C_{IgG3,FcRn}^{\alpha-PT} + C_{IgG4,FcRn}^{\alpha-PT}\big) + \Delta FcRn\Big]\frac{1}{v_{STB}} \quad (39)$$

$$\frac{dFc\gamma RIIb}{dt} = \Big[k_{off,1}(C_{IgG1,FFc\gamma RIIb} + C_{IgG1,Fc\gamma RIIb}^{\alpha-PT}) + k_{off,2}(C_{IgG2,Fc\gamma RIIb} + C_{IgG2,Fc\gamma RIIb}^{\alpha-PT}) + k_{off,3}(C_{IgG3,Fc\gamma RIIb} + C_{IgG3,Fc\gamma RIIb}^{\alpha-PT}) + k_{off,4}(C_{IgG4,Fc\gamma RIIb} + C_{IgG4,Fc\gamma RIIb}^{\alpha-PT}) - \big(k_{on,1}(IgG1_{STB} + IgG1_{STB}^{\alpha-PT}) + k_{on,2}(IgG2_{STB} + IgG2_{STB}^{\alpha-PT}) + k_{on,3}(IgG3_{STB} + IgG3_{STB}^{\alpha-PT}) + k_{on,4}(IgG4_{STB} + IgG4_{STB}^{\alpha-PT})\big)Fc\gamma RIIb_{STB} + k_t\big(C_{IgG1,Fc\gamma RIIb} + C_{IgG2,Fc\gamma RIIb} + C_{IgG3,Fc\gamma RIIb} + C_{IgG4,Fc\gamma RIIb} + C_{IgG1,Fc\gamma RIIb}^{\alpha-PT} + C_{IgG2,Fc\gamma RIIb}^{\alpha-PT} + C_{IgG3,Fc\gamma RIIb}^{\alpha-PT} + C_{IgG4,Fc\gamma RIIb}^{\alpha-PT}\big) + \Delta Fc\gamma RIIb\Big]\frac{1}{v_{STR}} \quad (40)$$

Together, this combined model consists of 62 equations.
